# Study protocol for the SOFIA project: Swallowing function, Oral health, and Food Intake in old Age: a descriptive study with a cluster randomized trial

**DOI:** 10.1186/s12877-017-0466-8

**Published:** 2017-03-23

**Authors:** Patricia Hägglund, Lena Olai, Katri Ståhlnacke, Mona Persenius, Mary Hägg, Maria Andersson, Susanne Koistinen, Eva Carlsson

**Affiliations:** 1 Oral and Maxillofacial Radiology, Department of Odontology, Faculty of Medicine, Umeå University, SE-90187 Umeå, Sweden; 2 School of Education, Health and Social Science, Dalarna University, Falun, Sweden; 3 Department of Public Health and Caring Sciences, Family Medicine and Preventive Medicine Section, Uppsala University, Uppsala, Sweden; 4 Public Dental Services, Örebro County Region, Örebro, Sweden; 5 Faculty of Health and Medicine, Örebro University, Örebro, Sweden; 6 Department of Health Science, Faculty of Health, Science and Technology, Karlstad University, Karlstad, Sweden; 7 Speech and Swallowing Centre, Department of Otorhinolaryngology, Hudiksvall Hospital, County Council of Gävleborg, Hudiksvall, Sweden; 8 Centre for Research and Development, Uppsala University, Uppsala, Sweden; 9 School of Health Sciences, Faculty of Health and Medicine, Örebro University, Örebro, Sweden; 10 University Health Care Research Center, Faculty of Health and Medicine, Örebro University, Örebro, Sweden

**Keywords:** Aged, Deglutition, Eating, Oral health, Quality of health care, Quality of life, Oral screen, Short-term care, Swallowing disorders

## Abstract

**Background:**

Extensive studies have shown that older people are negatively impacted by impaired eating and nutrition. The abilities to eat, enjoy food, and participate in social activities associated with meals are important aspects of health-related quality of life (HRQoL) and recovery after illness. This project aims to (*i*) describe and analyze relationships between oral health and oral HRQoL, swallowing ability, eating ability, and nutritional risk among older individuals admitted to short-term care; (*ii*) compare the perceptions that older individuals and staff report on care quality related to oral hygiene and eating; and (*iii*) study the feasibility and effects of a training program for people with impaired swallowing (i.e., dysphagia).

**Methods/Design:**

This project consists of two parts, which will be performed in five Swedish counties. It will include approximately 400 older individuals and 200 healthcare professionals. Part 1 is a cross-sectional, descriptive study of older people admitted to short-term care. Subjects will be assessed by trained professionals regarding oral health status, oral HRQoL, eating and nutritional risk, and swallowing ability. Swallowing ability will be measured with a teaspoon test and a swallowing capacity test (SCT). Furthermore, subjects and staff will complete a questionnaire regarding their perceptions of care quality.

Part 2 is a cluster randomized intervention trial with controls. Older participants with dysphagia (i.e., SCT <10 ml/s, measured in part 1) will be recruited consecutively to either the intervention or control group, depending on where they were admitted for short-term care. At baseline, all subjects will be assessed for oral health status, oral HRQoL, eating and nutritional risk, swallowing ability, and swallowing-related QoL. Then, the intervention group will receive 5 weeks of training with an oral screen for neuromuscular training focused on orofacial and pharyngeal muscles. After completing the intervention, and at six months post-intervention, all assessments will be repeated in both study groups.

**Discussion:**

The results will make important contributions to rehabilitation knowledge, including approaches for improving swallowing function, oral health, and food intake and for improving the quality of oral care for older people.

**Trial registration:**

This trial was retrospectively registered at ClinicalTrials.gov, on July 4, 2016, identifier: NCT02825927.

## Background

During the last 20–30 years, researchers from various disciplines have provided extensive knowledge about the negative consequences associated with impaired oral health, nutrition, and eating ability in older people. For example, these impairments can lead to slower wound healing and increase the risks of developing pneumonia and other infections, pressure ulcers, and fall injuries [1–3]. Furthermore, being able to eat, to enjoy food, and to participate in the social activities associated with meals are important aspects of the quality of life (QoL) for older people [4, 5]. One important aspect of eating is the ability to swallow safely. The prevalence of impaired swallowing (i.e., dysphagia) is high among older individuals; thus, this impairment has been described as a “geriatric giant” [6]. The prevalence of dysphagia in older populations has been reported to be 11–80% [6], with variations among studies that depended on the degree of frailty in the cohort, the presence of neurological disease, and the evaluation method used. Many older people adapt slowly to dysphagia by eating slower and changing food consistency; thus, many hold the opinion that developing dysphagia is a natural consequence of aging [7]. In recent years, more attention has been directed toward swallowing rehabilitation, and different treatment designs have been developed to improve swallowing function [8, 9]. One promising method of swallowing rehabilitation is training with an oral screen, which has been shown to improve dysphagia significantly [10]. Another important aspect of eating is oral health. Good chewing function requires a sufficient number of healthy teeth or functioning prostheses for adequate occlusion. Without adequate chewing, it is difficult to comminute and digest the food, which results in impaired swallowing [11]. Daily oral care is essential for good oral health. Older people that cannot perform oral care alone become dependent on health care professionals, which have differential knowledge about oral health, ambitions, and priorities [12].

When older people that are chronically ill undergo treatment for an acute illness or hospital treatment, they often recover for weeks or months in intermediate- or short-term care facilities that provide basic nursing care [13]. In Sweden, this type of care can be provided in a short-term care unit, a residential setting, or a hospital (for intermediate care). Short-term care can also include support for older people, when they are in frail conditions and are waiting for care-home placement, undergoing rehabilitation, require recurrent relief for family members that are informal caregivers, and even when they require end-of-life care [14, 15]. Although thousands of older people are admitted yearly to Swedish short-term care units, there is little knowledge about the content of care and the effects on individual health, functioning, and quality of life [14, 15]. We also lack knowledge on how caretakers perceive the quality of care that they deliver. The scarcity of empirical studies on short-term care might be related to the expected methodological and ethical problems. For example, problems may be caused by the short care interval (individuals are admitted for only a few weeks), the frail state of older individuals, and the complex care organization. It has been suggested that conducting research in a short-term care context poses challenges related to obtaining informed consent, achieving sufficient power in intervention studies, and controlling for confounding factors, such as the different skill mixes and staffing levels in various care units [16]. In summary, there is an urgent need for new knowledge concerning the content, quality, and effects of short-term care for older people.

## Methods

### Aims and research questions

The overall aims of this multidisciplinary, multicenter project are (*i*) to describe and analyze relationships between oral health and oral health-related quality of life (HRQoL), swallowing ability, eating ability, and nutritional risk for older individuals admitted to short-term care; (*ii*) to compare the perceptions reported by older individuals and staff on care quality related to oral hygiene and eating; and (*iii*) to study the feasibility and effects of a training program for older people that exhibit dysphagia, but have different diagnoses.

The research questions we will address are:

- What is the state of oral health among the older people admitted to short-term care?
- What is the state of swallowing ability among the older people admitted to short-term care?
- How do the older people admitted to short-term care describe their oral health and their oral HRQoL?
- How do the older people admitted to short-term care describe the quality of their oral care?
- How do staff members in the corresponding short-term care unit describe the quality of oral care delivered?
- Which factors impact the perceptions of care quality held by older individuals and the staff?
- What are the differences and similarities between older individuals and the staff in their perceptions of care quality?
- How does systematic training with an oral screen device for 5 weeks affect impaired swallowing function?
- How do older people admitted to short-term care describe their swallowing-related QoL?
- Is there any correlation between oral health, eating ability, and swallowing ability in older people admitted to short-term care?

This project will provide data for three doctoral theses; one student is a registered nurse, RN (MA); one is a registered dental hygienist, RDH (SK); and one is a speech-language pathologist, SLP (PH). Detailed descriptions of the specific aims and research questions for each doctoral project will be presented with the results in separate articles.

### Design and setting

The SOFIA project consists of two main parts; part 1 is a descriptive, cross-sectional study (aims *i* and *ii*), and part 2 is a cluster randomized, controlled trial (aim *iii*).

Data collection will take place in 32 short-term care units, located in five Swedish counties, in both rural and urban areas. The short-term care units are diverse in organization and staffing; they may be one section of a care home or an independent short-term care unit.

Participating short-term care units will be selected, based on informed consent from the heads of social welfare services and unit managers; the number of beds; the estimated number of discharges per month; and the geographic location. The sample in part 1 will comprise approximately 400 older participants. All participants will be assessed at baseline, and participants that exhibit low swallowing capacity (i.e., <10 ml/s) will be asked to participate in the interventional part of the study (part 2). In part 2, participating older persons will, after informed consent, be randomized into either the intervention or the control group, based on where they were admitted for short-term care. In addition to the baseline assessments, all participants in part 2 will be assessed after the intervention is completed (5 weeks) and 6-months post-intervention. The follow-up assessments will be conducted at the study participant’s location (e.g., in the short-term care unit, in the individual’s own home, or in a care home, where the older person resides at that time).

### Ethics

This project has been planned in accordance with the Helsinki Declaration [17], was approved by the Uppsala Regional Ethics Review Board, Sweden (Dnr 2013/100), and was retrospectively registered with Clinical Trials.gov, on July 4, 2016 identifier: NCT02825927.

### Participants

#### Aged participants

All individuals admitted to the selected short-term care units will be eligible for study participation. Inclusion criteria include: residence in the short-term care unit for at least three days, age ≥65 years, able to understand and speak Swedish, and cognitive capacity (judged by nurses) sufficient to give informed consent and to participate in data collection. Individuals that receive end-of-life care will be excluded.

The inclusion criteria for participation in part 2 include: dysphagia, displayed at baseline with a teaspoon test; and a rate of <10 ml/s on the Swallowing Capacity Test (SCT) [18].

Eligible participants will be asked to provide informed consent, in accordance with the research ethics requirements outlined in the Helsinki Declaration [17].

#### Care unit staff

All staff members working in the corresponding short-term care units will be eligible for study participation, including licensed practical nurses, nurse aides, registered nurses, occupational therapists, and managers.

All staff members will be informed about the study and will be provided with questionnaires at staff meetings. All questionnaires will be anonymized and mailed back to one of the researchers after review. A completed questionnaire will be taken to indicate informed consent. The manager of the short-term care unit will be instructed to remind the staff members to complete the questionnaire after two and four weeks.

### Data collection

Data collection in project parts 1 and 2 will be based on clinical assessments and self-reported measures, conducted by formally trained professionals (seven RDHs, and two doctoral students; with the professions RDH and SLP). These professionals have received specific training on data collection and on instructing participants and staff about the intervention; i.e., the swallowing training. The data collection period is 2013–2017.

In project part 1, clinical assessments will record: (a) eating ability and nutritional risk, (b) oral health status, (c) swallowing ability, (d) functional status (i.e., activities of daily living), (e) biometric measures (e.g., body weight and height), medical diagnoses, and planned discharge destinations, based on social service and nursing documentation. Self-reported measures will include: (f) care quality related to oral health and eating, (g) oral HRQoL, and (h) swallowing-related QoL. In project part 2, clinical assessments will record: (a) eating ability and nutritional risk, (b) oral health status, (c) swallowing ability, (d) functional status, and (e) biometrics and where the participant is located at the time. The self-reported measures will include: (g) oral HRQoL and (h) swallowing-related QoL. Table 1 provides an overview of the validated instruments used for the data collection in project parts 1 and 2, and the time points when the assessments will be performed. For detailed information about the instruments, see the primary references shown in Table 1.

**Table 1:** Instruments and measures to be implemented for data collection

Instrument	Reference	Outcome	Description	Time point
Katz Index of Activities of Daily Living (Katz-ADL)	[32, 33]	Functionality. Secondary outcome	Clinical assessment tool. Total score range: 1–7 (i.e., A-G), where 1 is “independent” and 7 is “very dependent”.	t1, t2, t3
Minimal Eating Observation and Nutrition Form –version II (MEONF-II)	[23, 24]	Risk of under-nutrition (UN), based on eating ability. Primary outcome	Clinical assessment tool. Total score range: 0 to 8, where 0–2 is no or low UN-risk; 3–4 is moderate UN-risk; ≥5 is high UN- risk.	t1, t2, t3
Quality of care from a patient’s perspective- modified version (QQP-modified version)	[19, 20]	Quality of care. Primary outcome	Self-reporting tool; 24 items. Each item is rated for both perceived reality (PR) and subjective importance (SI) with a 4-point Likert type scale. The PR range is: 1 (do not agree at all) to 4 (completely agree); the SI range is: 1 (little or no importance) to 4 (very highest importance). For each item, the PR and SI can also be rated ‘not applicable’ (58).	t1
Oral health status	Descriptive	Oral health.Primary outcome	Clinical assessment, which includes: an estimation of oral hygiene; a numeric registration of teeth; the presence of bridges, partial denture, full denture, implants; the number of occluding surfaces; and a record of the need for dental care. Also questions were asked about self-perceived oral health, whether there was an established dental contact, and the time since the most recent dental visit.	t1, t3
Revised Oral Assessment Guide (ROAG)	[25, 26]	Oral health status. Primary outcome	Clinical assessment tool, which includes 9 categories: voice, lips, mucous membranes, tongue, gums, teeth, dentures, saliva, and swallowing. Each category is described and graded on 3-point Likert scale: 1 indicates “healthy or normal condition”, 2 indicates “moderate alterations”, and 3 indicates “severe alterations”.	t1, t3
Oral Health Impact Profile (OHIP-14)	[28, 29]	Quality of life related to oral health. Secondary outcome	Self-reporting tool with 14 items in 7 categories. Each item is estimated on a 5-point Likert scale: 0 indicates “never” and 4 indicates “very often”.	t1, t3
The Swallowing Capacity Test (SCT)	[18]	Swallowing capacity. Primary outcome	A teaspoon test is carried out before the SCT. When signs of aspiration are observed, the SCT is not performed. Clinical assessment: the participant is instructed to drink 150 ml water from a glass as rapidly as possible, but safely, and to stop if any difficulty arises. Swallowing capacity is measured as the amount of water swallowed divided by the time it takes (ml/s). A capacity of ≥10 ml/s is considered normal. Signs of dysphagia will be recorded (e.g., coughing or wet/gurgling voice). When the subject fails the teaspoon test, a SCT score of 0 ml/s will be recorded.	t1, t2, t3
The Swallowing Quality of life Questionnaire (SWAL-QOL)	[30, 31]	Quality of life related to swallowing. Secondary outcome	Self-reporting tool with 44 items. Each item is rated on a 5-point Likert scale. Range: 1 “least favorable state” to 5 “most favorable state”. An additional 3 items are included for rating different types of food and drink consistencies and health status. These are rated on a 5-point Likert scale.	t1*, t2, t3

t1 = baseline; t1* = only older individuals with swallowing capacity <10 ml/s at baseline; t2 = immediately after the 5 week intervention; t3 = 6 months post-intervention

Data on external, objective care conditions, such as staffing, number of beds, length of stay, and prevalence of overcrowding, will be collected from the unit manager at each short-term care unit.

The nursing staff members’ perceptions of the quality of oral care and eating-related issues will be measured with a modified version of the Quality of care from a patient’s perspective questionnaire (QPP-Staff) [19, 20]. These data will be collected during a workplace meeting.

### Oral screen intervention

**Fig. 1:**
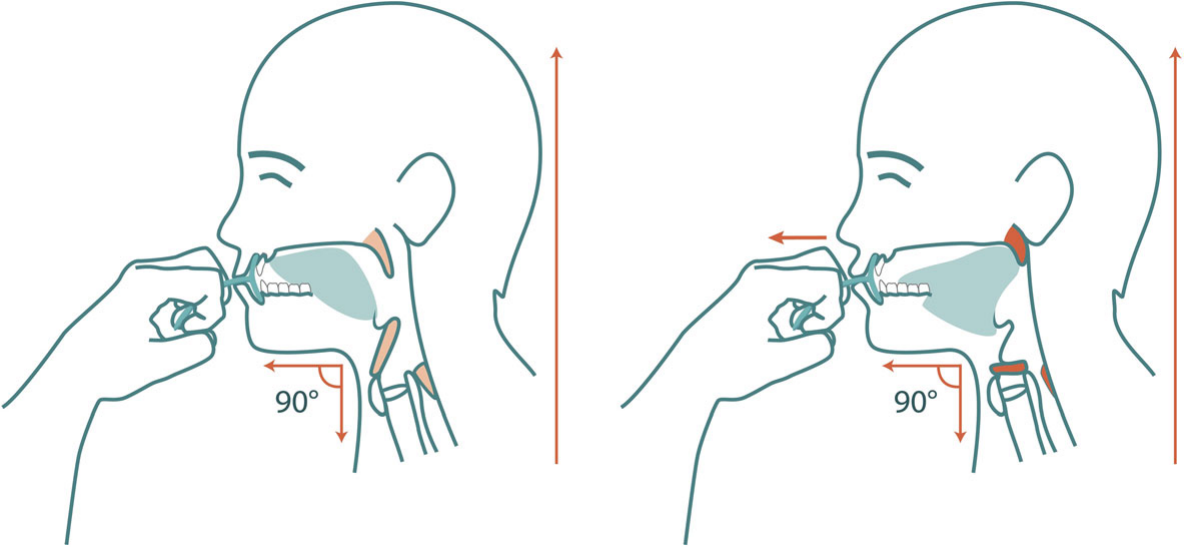
Oral screen training. (*Left*) The oral screen is inserted predentally, behind closed lips. (*Right*) The patient must press the lips firmly together, then strongly pull the handle straight forward, away from the mouth, and maintain pressure for 5–10 s. Illustrations: Mary Hägg©

We will use the IQoro® oral screen device for this study. This device is designed to strengthen the facial muscles [21], the oropharyngeal muscles, and the esophageal muscles [22]. Moreover, it increases activity in all muscles, from the lips down to the diaphragm [22]. The training protocol specifies using the device actively for 30 s, three times a day, before meals. Briefly, the oral screen is placed in the mouth, predentally, behind closed lips. The participant is asked to close the lips around the oral screen, to maintain it inside the lips; then, the participant pulls the handle horizontally, straight forward, as if to pull it out of the mouth, for approximately 5–10 s. The pulling force should be as high as possible without losing grip of the handle (see Fig. 1). The pulling maneuver is performed three times, with a 3-s rest between each maneuver [21]. When a participant cannot manage training independently (e.g., unable to close the lips or unable to pull the handle), a staff member is instructed to assist, with information about possible modifications in the training.

**Fig. 2:**
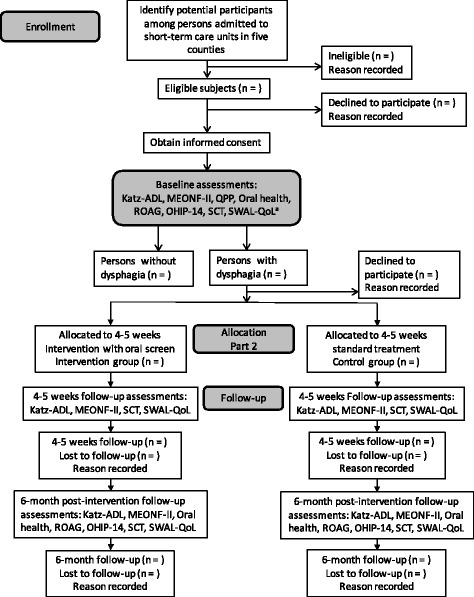
Flow-chart of the subject inclusion and data collection processes. ^a^ See Table 1

The participants in the intervention group will be asked to treat the dysphagia displayed at baseline by undergoing oral screen training for 5 weeks. The clinical assessments and self-reported measures (described above) will be collected at baseline, immediately after the intervention, and at 6 months post-intervention (see Fig. 2).

The control group will be asked to undergo the same assessments at the same times as the intervention group. Control participants will be given standard care (e.g., adjustments in food consistencies and posture instructions), according to the routines in the short-term care unit, where they reside. The data collection process is described chronologically in Fig. 2.

### Sample size calculation

In study 2, the primary outcome variables will be the changes in swallowing capacity (based on the SCT), measured at the start and end of treatment, and at follow-up, 6 months later. The sample size calculation will be based on the assumption that the data will be normally distributed. To retain an overall type I error of 5%, the type I error will be set to 2.5% for the primary endpoint. Based on historical data, we assume that the standard deviation of the change in SCT level will be 3 ml/s. To detect a critical difference of 2.8 ml/s in swallowing capacity between the intervention and control groups with a power of 80% and type I error of 2.5%, a sample size of at least 22 subjects would be required to fulfill the study protocol in each group. The total number of subjects included must be somewhat higher, to compensate for drop-outs.

### Statistical methods

The statistical package, IBM/SPSS®, will be used to perform all data analyses. *P*-values less than 0.05 will be considered statistically significant. Descriptive statistics will be used to describe eating ability, oral health status, swallowing ability, quality of oral care, and swallowing-related QoL, among older individuals admitted to short-term care units. Descriptive statistics will also be used to describe the staffs’ perceptions of care quality. Univariate, bivariate, and multivariable regression (linear and logistic) analyses will be used to evaluate the associations between various dependent and independent variables. The Bonferroni correction will be used when multiple, pair-wise tests are performed on a single set of data, to reduce the chances of obtaining type I errors. Continuous variables will be presented as the mean value and standard deviation; categorical variables will be reported as the median and interquartile range. Differences between groups (e.g., intervention vs. control, older individuals vs. staff) will be assessed with independent sample t-tests (for continuous variables), chi-square tests (for categorical variables), and ANOVA or Kruskal-Wallis (for more than 2 groups).

## Discussion

This present protocol paper describes the design of a multidisciplinary, multicenter study, focused on Swedish short-term care, and we include a second, randomized case-controlled interventional study. This project has two important aims. First, it aims to describe important aspects of oral health, swallowing ability, and eating ability in older individuals, and to investigate the feasibility and effects of a program of oral screen training for dysphagia. Second, the project aims to study the quality of oral health-related care, as perceived by older individuals and staff in the short-term care units. The results of this project will provide important new knowledge for the development of short-term care for older individuals, in this specific care context, which has not been explored before. These resulting descriptions of the strengths and weakness in the quality of oral care will provide a basis for establishing guidelines for improving oral care in short-term care units. Moreover, in this context, the results of the swallowing training program with an oral screen will indicate whether this program will promote improvements in short-term care for this older population.

### Methodological considerations

Methodological and ethical problems were previously reported as challenges that explained the lack of empirical studies conducted in the short-term care context [16]. Therefore, the present project includes several strategies to increase the probability of succeeding and to strengthen the results, as follows: (1) only participants with sufficient cognitive capacity are included; (2) older individuals in end-of-life care are excluded; (3) multiple short-term care units are included, housed in various settings and located in different parts of Sweden; (4) a cluster randomization trail design is implemented; (5) both clinical and self-reported assessments are considered; (6) the data collectors are professionals that hold relevant clinical experience in communicating with older individuals and assessing their oral health and swallowing ability; in addition the research team has experience in the care context, where the data will be collected.

By minimizing methodological and ethical challenges, our results, regarding oral health status, swallowing, eating ability, and nutritional risk, might not be representative of all older people in short-term care, e.g., individuals in the highest risk categories will not be included. Other methodological challenges might arise when performing the intervention study with late follow-up assessments (six months). These challenges include accessing information on where the participant is located at the time of the follow-up; managing the high risk of drop-out, due to mortality, in the study population; and recruiting for part 2 of the study. The latter problem is expected, because older people might have low motivation to participate in swallowing rehabilitation, due to fatigue, or because they have adapted to their situation and find no use in training [6].

### Measurement tools

The minimal eating observation and nutrition form - version II (MEONF-II) [23, 24] is an easy-to-use, rapid, sensitive, screening tool that allows substituting of body mass index (BMI) with calf circumference, which increases its usability in this context, for assessing the risk of undernutrition. However, completing the form might be time-consuming for the nursing staff, and it requires a certain degree of familiarity with the older person.

The revised oral assessment guide (ROAG) [25, 26] is a systematic assessment tool for detecting problems related to teeth and dentures in older individuals. It was designed to be used by nursing staff, and it is well-documented in Sweden, because it is included in Senior Alert, the national registry of quality in geriatric care [27]. However, the assessment is performed solely with a mouth mirror and flashlight, not in a dental clinic.

The SCT [18] is a simple, easy-to use screening test for determining the risk of dysphagia in a non-hospital context. The SCT is a feasible tool for evaluating training paradigms for treating dysphagia [8]. The SCT requires an evaluation of age as a covariate, when performing data analysis.

Several self-assessment questionnaires (Table 1) will be used in the present study to evaluate QoL related to different health areas, including: the OHIP-14 [28, 29], the QPP-patient [19, 20], and the SWAL-QOL [30, 31]. These questionnaires will contribute multifaceted information from the perspectives of older individuals. The results will guide further improvements in QoL in the short-term care context. However, answering questionnaires might be exhausting for older people. Therefore, there might be a risk of bias, if older individuals require support in reading and interpreting the questions. However, this type of support might lead to more accurate responses and fewer drop-outs.

The QPP-staff is a modified version of the QPP-patient questionnaire [19, 20]. The QPP-staff will provide important knowledge, from the perspective of nursing staff members, about the quality of care for older individuals in short-term care units. The challenges for this instrument are the risks of hasty, incomplete responses and drop-outs among the nursing staff members expected to complete the questionnaires. Due to the working situations of nursing staff, the many other tasks and priorities related to patient care might compromise their compliance with study requirements.

The Katz-ADL instrument [32, 33] has been used in geriatric care since the 1960s. It is a simple tool for assessing functional status in older individuals. It enables evaluations of rehabilitation during follow-up and the prognosis of recovery after an illness. This tool is sensitive to broad changes in declining health status; however, it can be limited in its ability to measure small improvements in rehabilitation.

If our findings show that the swallowing intervention could be useful in this context (older individuals with various diagnoses complicated with dysphagia), the training method should be implemented as a treatment option for all older individuals with dysphagia. The staff in all types of geriatric care should be educated in implementing this method. Additionally, a successful swallowing intervention would provide benefits beyond the eating and swallowing abilities of the individual, because it would also reduce morbidity and mortality. We expect that a better understanding of oral health in the older population studied will highlight the unaddressed needs related to oral care and malnutrition. Moreover, our findings will provide important quality indicators of good oral health and eating-related nursing care, in short-term care units.

## Abbreviations

HRQoL: Health-related quality of life
Katz-ADL: Katz activity of daily living
MEONF-II: Minimal eating observation and nutrition form –version II
OHIP-14: Oral health impact profile
QoL: Quality of life
QPP: Quality of care from a patient perspective
RDH: Registered dental hygienist
ROAG: Revised oral assessment guide
SCT: Swallowing capacity test
SLP: Speech-language pathologist
SWAL-QoL: Swallowing quality of life questionnaire
